# Impact of psychological inflexibility on depressive symptoms and sleep difficulty in a Japanese sample

**DOI:** 10.1186/s40064-016-2393-0

**Published:** 2016-06-14

**Authors:** Tsukasa Kato

**Affiliations:** Development of Social Psychology, Toyo University, 5-28-20, Hakusan, Bunkyo-ku, Tokyo, 112-8606 Japan

**Keywords:** Psychological inflexibility, Acceptance and commitment therapy, Depressive symptoms, Mindfulness, Insomnia, Acceptance and Action Questionnaire

## Abstract

**Background:**

Psychological inflexibility is a core concept in the acceptance and commitment therapy (ACT) model. The current study hypothesized and tested two models in which psychological inflexibility was linked with higher levels of depressive symptoms and sleep difficulty.

**Results:**

To attain data, Japanese university students (*N* = 633) completed questionnaires related to psychological inflexibility, depressive symptoms, and sleep difficulty. Psychological inflexibility was significantly correlated with higher levels of both depressive symptoms (*r* = 0.61) and sleep difficulty (*r* = 0.39). Structural equation modeling showed that psychological inflexibility was associated with higher levels of depressive symptoms after controlling for the effect of sleep difficulty. Additionally, psychological inflexibility was correlated with higher levels of sleep difficulty after controlling for the effect of depressive symptoms. These findings might assist with motivating clinicians to use ACT for insomnia.

**Conclusions:**

The current study found that greater psychological inflexibility was linked with high levels of depressive symptoms and sleep difficulties. These findings might assist with motivating clinicians to use ACT for insomnia.

## Background

Psychological inflexibility is defined as “the rigid dominance of psychological reactions over chosen values and contingencies in guiding action” (p. 678, Bond et al. 2011), which often occurs when individuals attempt to avoid experiencing unwanted internal events. Meanwhile, psychological flexibility refers to one’s ability to focus on the present moment fully and, according to what the situation affords, change or persist with behavior in the pursuit of goals and values (Hayes et al. 2006). Psychological inflexibility is a core concept in the acceptance and commitment therapy (ACT) model (Hayes et al. 1999, 2006), which hypothesizes that psychological inflexibility leads to increased psychological dysfunction and reduced quality of life. Thus, an important goal of ACT is to increase psychological flexibility.

Psychological inflexibility can serve as a risk factor for several disorders including insomnia (Hayes et al. 2006) and depression (Chawla and Ostafin 2007; Ruiz 2010). Insomnia and depression are highly prevalent disorders. An epidemiological study (Kim et al. 2000) in Japan reported its one-month prevalence of insomnia to be approximately 21.4 %. Since sleep difficulty is a primary indicator of insomnia (National Institutes of Health (NIH) 1998), the present study focused on this as a key insomnia-related symptom. Meanwhile, in Japan, the lifetime prevalence of major depressive episodes is 6.6 % (SE = 0.5) (Kessler and Bromet 2013). The current study focused on psychological inflexibility, and examined the relationships between psychological inflexibility, depressive symptoms, and sleep difficulty.

### Psychological inflexibility and depressive symptoms

The efficacy of ACT-based treatment has been supported by a growing body of empirical evidence (for reviews, see Hayes et al. 2006; Ruiz 2010, 2012; Öst 2008, 2014), which suggests that by increasing psychological flexibility, depression is lowered (for reviews, Chawla and Ostafin 2007; Ruiz 2010). Psychological flexibility is established through six underlying processes, including attempting to embrace unwanted feelings and thoughts instead of avoiding, distracting, or ignoring them (acceptance); attempting to obtain distance from feelings and thoughts by changing undesirable thoughts and negative private events (cognitive defusion); and attempting to perceive a transcendent sense of self (self as context) (Hayes et al. 2006).

In the ACT model, depressive symptoms reflect unwanted internal experiences (Hayes et al. 2006), and depressed individuals have inflexible cognitive thoughts and feelings, as well as various avoidant behaviors to reduce unwanted internal experiences (Williams 2008). These negative response patterns in depressive symptoms are also identified as negative attributional styles in hopelessness theory (Alloy et al. 1988), ruminative responses in the response style theory (Nolen-Hoeksema, 1991), and negative mood regulation (Catanzaro and Mearns 1990). The ACT model hypothesizes that these negative response patterns are improved by promoting psychological flexibility (Hayes et al. 2006), and previous research has provided strong evidence that greater psychological inflexibility is associated with more depressive symptoms among college students (e.g., Gloster et al. 2011; Masuda et al. 2014; Woodruff et al. 2014), employees (e.g., Bond et al. 2011; Gloster et al. 2011), and individuals with panic disorders (e.g., Gloster et al. 2011; Kämpfe et al. 2012), anxiety disorders (e.g., Curtiss and Klemanski 2014; Fergus et al. 2013), and chronic pain (e.g., McCracken et al. 2011, 2014). A meta-analytic study (Ruiz, 2010) showed that the weighted correlation for psychological inflexibility and depressive symptoms was 0.55 (*N* = 3323), and correlations ranged from 0.37 to 0.77. An intervention study (Bohlmeijer et al. 2011) in which ACT was used to treat depressive symptomatology suggested that improved psychological inflexibility scores (post-treatment minus baseline scores) were related to lower depressive symptoms scores at follow-up after controlling for the effects of baseline psychological inflexibility and depressive symptoms scores.

### Psychological inflexibility and insomnia

In addition to depressive symptoms, insomnia symptoms also contribute to psychological distress and functional impairment; thus, the ACT model hypothesizes that insomnia symptoms, including sleep difficulties, may also be ameliorated by promoting psychological flexibility (Hayes et al. 2006). Recently, mindfulness and acceptance-based approaches—which are some of the processes used to increase psychological flexibility in the ACT model (Hayes et al. 2006)—have been proposed to address the cognitive mechanisms of the development and maintenance of insomnia, including sleep difficulties (e.g., Lundh 2005; Ong et al. 2012). According to Lundh (2005), insomnia symptoms result from an interaction between sleep-interfering processes (i.e., arousal-producing processes that interfere with sleep) and dysfunctional sleep-interpreting processes (i.e., misperceptions of sleep). Sleep-interpreting processes—for example, dysfunctional beliefs, expectations, and attributions concerning sleep, and the causes and consequences of poor sleep—may be improved by mindfulness and acceptance-based treatments, which cultivate acceptance of spontaneously occurring physical and psychological experiences that precede sleep onset.

Ong et al. (2012) proposed a two-level model of sleep-related cognitive arousal consisting of primary and secondary arousal. Secondary arousal, which directly relates to mindfulness and acceptance-based approaches for insomnia, involves dysfunctional thoughts about sleep and has four elements: attention and emotional bias toward sleep-seeking or sleep-aversive thoughts and behaviors, rigidity in sleep-related behaviors and beliefs, attachment to sleep-related needs and expectations, and absorption in solving the sleep problems (Ong et al. 2012). Secondary arousal is improved by shifting these elements to adaptive stances through mindfulness and acceptance-based treatments. Namely, Ong et al. (2012) and Lundh’s (2005) studies emphasize the importance of cognitive processes (i.e., sleep-interpreting processes and secondary arousal, respectively) in the development and maintenance of insomnia, and suggest that the cognitive processes may be improved by mindfulness and acceptance-based treatments for insomnia (i.e., by fostering psychological flexibility). In fact, several studies have provided evidence that mindfulness and acceptance-based treatments might improve insomnia and sleep quality (for a review, Winbush et al. 2007). Therefore, it is considered that greater psychological inflexibility is associated with more insomnia symptoms.

Evidence for the efficacy of mindfulness and acceptance-based treatments for insomnia symptoms has been provided by several studies (e.g., Garland et al. 2015; Gross et al. 2011; Heidenreich et al. 2006; McCracken et al. 2011; Ong et al. 2008; Yook et al. 2008). However, few studies (e.g., McCracken et al. 2011) have examined the association between psychological inflexibility and insomnia, including sleep difficulties. One study involving a sample of adult patients with chronic pain indicated that psychological inflexibility was positively correlated with insomnia severity and problems with sleep (McCracken et al. 2011). The current study hypothesized that psychological inflexibility would be associated with greater sleep difficulty, based on mindfulness and acceptance-based approaches to insomnia (e.g., Lundh 2005; Ong et al. 2012). The sample in McCracken et al.’s study (2011) was comprised of adults with chronic pain, whereas our sample was comprised of college students. In their study, psychological inflexibility was a predictor for insomnia, while a variable related to depressive symptoms was not assessed.

### Role of psychological inflexibility in the association with depressive symptoms and insomnia symptoms

Previous studies have provided strong evidence of the relationship between insomnia and depression, including studies of younger adults or adolescents (e.g., Roberts and Duong 2013) and large-scale studies of the Japanese population (e.g., Yokoyama et al. 2010). However, both the clinical and theoretical relationships between depression and insomnia are complex. Staner (2010) suggests the possibility that depression and insomnia are truly comorbid and related either causally or otherwise. Additionally, some studies suggest that depressive symptoms are one of the most common risk factors for insomnia (for reviews, see Berk 2009; Staner 2010). This is posited because insomnia is included as one of the diagnostic criteria for major depressive episodes in several editions of the Diagnostic and Statistical Manual of Mental Disorders (DSM). Meanwhile, other studies suggest that insomnia is a risk factor for depressive symptoms (for reviews, see Baglioni et al. 2011; Cole and Dendukuri 2003; Franzen and Buysse 2008).

Some pharmacological and nonpharmacological interventions for insomnia may lessen depression severity and hasten recovery (for a review, Franzen and Buysse 2008). In fact, cognitive-behavioral therapy for insomnia reduced depressive symptoms as well as insomnia symptoms in individuals with major depressive disorders (e.g., Manber et al. 2008; Taylor et al. 2007). Likewise, mindfulness and acceptance-based treatment for insomnia, with an emphasis on the acceptance of unwanted feelings and thoughts, attenuated depressive symptoms as well as sleep problems (e.g., Yook et al. 2008). Moreover, evidence have been provided that mindfulness and acceptance-based treatments for depressed patients improved sleep quality (e.g., Biegel et al. 2009; Schramm et al. 2016). For example, mindfulness-based cognitive therapy for adolescent psychiatric outpatients, including depressed patients, improved self-reported sleep quality as well as depressive mood more than individual or group therapy and/or psychotropic medication management (Biegel et al. 2009). These findings suggest that interventions for insomnia may alleviate insomnia symptoms with the result that depressive symptoms are attenuated, or that interventions for insomnia may reduce depressive symptoms with the result that insomnia symptoms are attenuated. The former may imply that insomnia mediates the relationship between psychological inflexibility and depressive symptoms, while the latter may imply that depressive symptoms mediate the relationship between psychological inflexibility and insomnia.

**Fig. 1:**
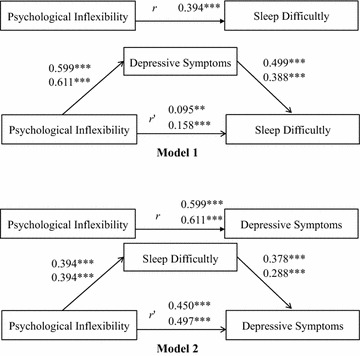
The Models for psychological inflexibility, depressive symptoms, and sleep difficulty. For clarity, error terms for each indicator variable were omitted from the diagram. The *upper values* are the coefficients when the Patient Health Questionnaire-9 (PHQ) was used to measure depressive symptoms, and the *bottom values* are the coefficients when the 8-item PHQ was used to measure depressive symptoms. *N* = 663. ***p < .001; ** *p* < .01

Therefore, the current study proposed two models (see Fig. 1): in Model 1, depressive symptoms mediate the relationship between psychological inflexibility and sleep difficulty; in Model 2, sleep difficulty mediates the relationship between psychological inflexibility and depressive symptoms. The current study hypothesized that psychological inflexibility would be associated with higher levels of depressive symptoms and sleep difficulty in both models.

## Method

### Participants and procedure

Participants in this study were 663 college students in Japan. Participants included 288 men and 375 women between the ages of 18 and 26 years (*M* = 19.60, *SD* = 1.10) who were recruited from introductory psychology classes. All participants were born in Japan and indicated that their ethnicity was Japanese. After giving informed consent, they completed a set of questionnaires. The participants received course credit for their participation. All procedures followed were in accordance with the ethical standards of the responsible committee on human experimentation (institutional and national) and with the Helsinki Declaration of 1975, as revised in 2000. Written informed consent was obtained from all participants before they were included in the current study.

### Measures

All measures that were originally written in English were independently translated into Japanese by three native Japanese psychologists. They were then back-translated into English by a native English psychologist. After the back translation, the original and back-translated questionnaires were compared for discrepancies. Modifications were made to the translated questionnaires after discussions were held between the translators.

#### Psychological inflexibility

The 7-item Acceptance and Action Questionnaire-II (AAQ-II; Bond et al. 2011) was used to measure psychological inflexibility (sample items: “my painful memories prevent me from having a fulfilling life” and “emotions cause problems in my life”). The alpha coefficients for the AAQ-II ranged from 0.78 to 0.88 across six samples (Bond et al. 2011). Furthermore, the 3- and 12-month test–retest reliability coefficients were 0.81 and 0.79 over the two periods, respectively (Bond et al. 2011). The AAQ-II was developed in the United States and the United Kingdom (Bond et al. 2011), and has been translated into several languages such as Italian (e.g., Pennato et al. 2013) and German (e.g., Gloster et al. 2011). A study using an Asian sample (Zhang et al. 2014) reported Cronbach’s alphas of the AAQ-II as 0.86 and 0.88 and revealed that AAQ-II scores were associated with higher levels of depressive symptom, negative affect, and trait anxiety. The mean scores of the AAQ-II among undergraduate students in the United Kingdom and China were 17.34 (*SD* = 4.37, *N* = 433, Bond et al. 2011) and 23.83 (*SD* = 6.72, *N* = 366, Zhang et al. 2014), respectively.

In the present study, participants were asked to rate each item on a 7-point Likert scale ranging from 0 (*never true*) to 6 (*always true*). The instruction was as follows: “Below you will find a list of statements. Please rate the truth of each statement as it applies to you. Use the following scale to make your choice.” In order to test the unidimensional structure of the AAQ-II items, as reported by Bond et al. (2011), a confirmatory factor analysis was conducted with a maximum likelihood method using our data in this study. Two error terms were allowed (Items 1 and 4; Items 3 and 5). According to the guidelines suggested by Hu and Bentler (1999), the root mean squared error of approximation (0.053) and standardized root mean square residual (0.019) values for the one-factor structure were less than the cut-off criteria of 0.06 and 0.08, respectively, and the comparative fit index value (0.991) was greater than the cutoff criterion of 0.90, which indicated acceptable fit.

#### Depressive symptoms

Depressive symptoms were measured using the 9-item Patient Health Questionnaire (PHQ-9; Kroenke et al. 2001), which is based upon the DSM-IV criteria for major depression. The validity and reliability of the PHQ-9 has also been established in a number of non-English-speaking populations (for recent reviews, see Manea et al. 2012, 2015), including Japan (e.g., Inagaki et al. 2013; Muramatsu et al. 2007). In the current study, scores for both an 8-item version and the PHQ-9 were computed. In the former scale, Item 3 (*trouble falling or staying asleep, or sleeping too much*) was deleted from the PHQ-9 because one purpose of this study was to examine the relationships between depressive and insomnia symptoms. As mentioned in the Introduction, insomnia constitutes one of the DSM-IV’s diagnostic criteria for major depression, and this item only partially reflects insomnia symptoms. The current study used the PHQ-9 as well because information from the PHQ-9, which has been used frequently in published studies to measure depressive symptoms (Manea et al. 2012, 2015), would be helpful for interpreting our findings in the current study.

Total scores on the PHQ-9 range from 0 to 27, and scores of 5, 10, 15, and 20 from the PHQ-9 indicate thresholds of mild, moderate, moderately severe, and severe depression symptoms, respectively (Kroenke et al. 2001). Other researchers (e.g., Manea et al. 2012, 2015) recommend a PHQ-9 score of 10 or greater as the superior cut-off point for this scale. In addition, the measure had a sensitivity of 0.98 and a specificity of 0.55 for major depression for outpatients in Japan (Inagaki et al. 2013). The mean score of the PHQ-9 for college students in Japan was 6.18 (*SD* = 4.96, *N* = 160, Hirao 2015). In the current study, participants rated each item according to their experiences within the last two weeks on a 4-point Likert scale ranging from 0 (*not at all*) to 3 (*nearly every day*).

#### Sleep difficulty

The Sleep Difficulty Scale (SDS; Kato 2014a) was used to measure sleep difficulty. The 4-item SDS is a subscale of the Sleep Quality Questionnaire (Kato 2014a), and was designed to measure the core features of insomnia based on the NIH’s definitions (1998). Additionally, this scale is comprised of brief items to which Japanese participants can easily respond. For example, Japanese people reported that some items of the Pittsburgh Sleep Quality Index (Buysse et al. 1989), which is the most commonly used scale for sleep quality, were difficult to understand or answer due to differences between Western and Japanese cultures (Doi et al. 1998), but not due to it being translated into Japanese. The Japanese version of the SDS has been confirmed as psychometrically sound with a 0.79 test–retest reliability coefficient over an 8-week period and a Cronbach’s alpha coefficient of 0.74 in college student samples (Kato 2014a). Additionally, as measured by other insomnia scales, SDS scores have been associated with sleep latency and insomnia symptoms. Furthermore, these scores have been reported as related to higher levels of psychological distress, depressive symptoms, and functional and behavioral impacts of fatigue (Kato 2014a). Another study in Japan (Kato 2014b) also showed that SDS scores were associated with depressive symptoms, after controlling for the effects of suicidal ideation. The mean score of the SDS among undergraduate students in Japan was 7.14 (*SD* = 3.83, *N* = 716, Kato 2014a). In the current study, participants responded to items about their usual sleep habits in the last 2 weeks. They rated the extent of agreement with each item on a 5-point Likert scale, ranging from 0 (*strongly disagree*) to 4 (*strongly agree*).

### Data analysis

Structural equation modeling (SEM) analysis was conducted using maximum likelihood estimation in order to test our two proposed models. According to Baron and Kenny (1986), four conditions are necessary to establish mediation, including a significant (a) relationship between the predictor and outcome, (b) relationship between the predictor and mediator, (c) relationship between the mediator and outcome, and (d) reduction in the strength of the relationship between the predictor and outcome when the mediator is added to the model. Mediating effects of depressive symptoms or sleep difficulty were examined using a combination procedure based on MacKinnon and colleagues’ study (MacKinnon et al. 2002). First, a difference in coefficients (path *r* minus path *r*′) was tested using Freedman and Schatzkin’s (1992) method (see Fig. 1). Second, in addition to Sobel tests (1982), the bias-corrected bootstrapping method for indirect effects was computed. If the 95 % confidence interval (CI) estimate excluded zero, we concluded that the test indicated a significant indirect effect. This method is more statistically appropriate than alternative tests of significance of indirect effects (e.g., the Sobel test) in that it does not assume a normal distribution of indirect effect sizes (MacKinnon et al. 2000). The statistics of the Sobel test were provided as they have traditionally been used as indicators of mediating effects.

## Results

Regarding the characteristics of depressive symptoms in our sample, the percentage of scores that were ≥5, ≥10, ≥15, and ≥20 on the PHQ-9 were 58.82 % (95 % CI [55.51, 62.75]), 24.59 % (95 % CI [21.42, 27.90]), 7.99 % (95 % CI [6.03, 10.11]), and 2.41 % (95 % CI [1.36, 3.62]), respectively. The means, standard deviations, alpha coefficients, and correlations of all variables are shown in Table 1.

**Table 1:** Means, standard deviations, alpha coefficients and correlation matrix for all variables

	Variable	2	3	4	*Mean*	*SD*	α
1	Psychological Inflexibility	.599***	.611***	.394***	17.75	8.95	.887
2	Depressive symptoms (9-item)		.983***	.556***	6.59	4.97	.819
3	Depressive symptoms (8-item)			.484***	5.62	4.36	.806
4	Insomnia symptoms				6.90	3.92	.733

*N* = 663

*** *p* < .001

### Model 1

All correlation coefficients were significant at *p* < 0.001. As predicted, psychological inflexibility scores were significantly correlated with higher PHQ (for both PHQ-9 and 8-item PHQ) scores and SDS scores. SEM results are shown in Fig. 1. The direct effect of psychological inflexibility on sleep difficulty was significant (*β* = 0.095, *p* < 0.01) when the PHQ-9 scores were added to the model, as were the differences between path *r* and path *r*′ (*t*(661) = 27.44, *p* < 0.001) and the indirect effect of psychological inflexibility on sleep difficulty via depressive symptoms (*β* = 0.299; Sobel test, *z* = 10.42, *p* < 0.001). Because the 95 % CI [0.110, 0.158] using the bias-corrected bootstrapping method excluded zero, the pathways between psychological inflexibility and depressive symptoms (*β* = 0.599, *p* < 0.001) and between depressive symptoms and sleep difficulty (*β* = 0.499, *p* < 0.001) were also significant.

When the 8-item PHQ scores were added to the model, the direct effect of psychological inflexibility on sleep difficulty was significant (*β* = 0.158, *p* < 0.001), as were the differences between path *r* and path *r*′ (*t*(661) = 20.26, *p* < 0.001) and the indirect effect of psychological inflexibility on sleep difficulty via depressive symptoms (*β* = 0.237, *z* = 8.27, *p* < 0.001). The pathways between psychological inflexibility and depressive symptoms (*β* = 0.611, *p* < 0.001) and between depressive symptoms and sleep difficulty (*β* = 0.388, *p* < 0.001) were also significant because the 95 % CI [0.083, 0.130] excluded zero.

These results indicated that depressive symptoms mediated psychological inflexibility and sleep difficulty. Moreover, they revealed that, after controlling for the effect of depressive symptoms, greater psychological inflexibility was associated with higher levels of sleep difficulty.

### Model 2

The direct effect of psychological inflexibility on depressive symptoms was significant (*β* = 0.450, *p* < 0.001) when the PHQ-9 scores were added to the model. Additionally, the indirect effect of psychological inflexibility on depressive symptoms via sleep difficulty (*β* = 0.149, *z* = 8.24, *p* < 0.001) and the differences between path *r* and path *r*′ (*t*(661) = 21.79, *p* < 0.001) were significant. Because the 95 % CI [0.065, 0.104] excluded zero, the pathways between psychological inflexibility and sleep difficulty (*β* = 0.394, *p* < 0.001) and between sleep difficulty and depressive symptoms (*β* = 0.378, *p* < 0.001) were also significant.

When the 8-item PHQ scores were added, the direct effect of psychological inflexibility on depressive symptoms was significant (*β* = 0.497, *p* < 0.001). In addition, the indirect effect of psychological inflexibility on depressive symptoms via sleep difficulty (*β* = 0.113, *z* = 7.02, *p* < 0.001) and the differences between path *r* and path *r*′ (*t*(661) = 18.90, *p* < 0.001) were significant. The pathways between psychological inflexibility and sleep difficulty (*β* = 0.394, *p* < 0.001) and between sleep difficulty and depressive symptoms (*β* = 0.288, *p* < 0.001) were significant because the 95 % CI [0.043, 0.072] excluded zero.

Thus, psychological inflexibility was significantly associated with depressive symptoms via sleep difficulty. Furthermore, after controlling for the effect of sleep difficulty, greater psychological inflexibility was associated with higher levels of depressive symptoms.

## Discussion

Based on previous ACT studies, the current study hypothesized that greater psychological inflexibility would be associated with higher levels of depressive symptoms and sleep difficulty. As expected, greater psychological inflexibility was significantly correlated with higher depressive symptoms and sleep difficulty, and these correlations resulted in medium-to-large effect sizes. Specifically, the correlation coefficient (*r* = 0.60) between psychological inflexibility and depressive symptoms in our sample was close to the weighted correlation of 0.55 found in a previous meta-analytic study (Ruiz 2010).

While it is well known that greater psychological inflexibility is associated with more depressive symptoms, insufficient research exists into the relationship between psychological inflexibility and insomnia symptoms. The current study hypothesized that greater psychological inflexibility would be associated with more sleep difficulty, based on the ACT model and mindfulness and acceptance-based approaches to insomnia. The sample in McCracken et al.’s study (2011), which examined the relationships between psychological inflexibility and insomnia, was comprised of adults with chronic pain, whereas our sample was comprised of college students. The correlations for McCracken et al. (2011) and the current study were 0.28 (*N* = 159) and 0.39 (*N* = 663), respectively; the difference was not significant (*z* = 1.45, *p* = 0.148).

The current study presented and tested two models of the relationships between psychological inflexibility, depressive symptoms, and sleep difficulty. In both Models 1 and 2, the mediation model satisfied Baron and Kenny’s (1986) four conditions for mediation when both the 8-item PHQ and PHQ-9 scores were used. The Model 1 results suggested that greater psychological inflexibility was linked with increased insomnia symptoms via depressive symptoms, and the Model 2 results showed that sleep difficulty mediated the relationship between psychological inflexibility and depressive symptoms.

The findings that mindfulness and acceptance-based treatments for depressed patients improved sleep quality (e.g., Biegel et al. 2009), and that mindfulness and acceptance-based treatments for insomnia alleviated depressive symptoms (e.g., Yook et al. 2008), may help when interpreting the mediation models proposed in the current study. The former and latter findings may support our results of the mediations in Models 1 and 2, respectively. However, studies that examine the efficacy of mindfulness and acceptance-based treatments for insomnia attenuating depressive symptoms, or mindfulness and acceptance-based treatments for depression improving insomnia, are scant. Therefore, further studies of mindfulness and acceptance-based treatments for insomnia or depression that examine their effects on depressive symptoms and insomnia may help to understand the role of psychological inflexibility in association with depressive symptoms and insomnia symptoms. In addition, studies that examine parallel or sequential mediators using longitudinal designs may help to elucidate their associations.

However, it is important to note that our data were obtained using a cross-sectional design; thus, sufficient causal evidence for a relationship between depressive symptoms and sleep difficulty, as well as between psychological inflexibility, depressive symptoms, and sleep difficulty, could not be provided. In particular, the causal relationship between depressive symptoms and sleep difficulty is quite complex (Staner 2010), although the relationship has been discussed in the epidemiological and diagnostic literature. Therefore, the primary focus of the current study was not to examine the causal relationship between depressive symptoms and sleep difficulty, but rather the relationship between psychological inflexibility and either depressive symptoms or sleep difficulty. Our results merely suggested that greater psychological inflexibility was associated with higher levels of depressive symptoms and sleep difficulty; however, our findings may contribute to the development of new applications of ACT, particularly for insomnia.

### Limitations

The current study has some limitations. First, as mentioned above, causality could not be established because of the cross-sectional design. Thus, future research should use a prospective design or test the effects of an intervention that ameliorates psychological inflexibility (e.g., based on ACT) to examine the relationships between psychological inflexibility and depressive and insomnia symptoms. Second, self-report cross-sectional data are subject to biases due to common method variance (CMV). However, the current study used many of the procedures suggested by Podsakoff et al. (2012) to control for common method biases, including protecting respondent anonymity, reducing evaluation apprehension, and using reliable and valid measures for each construct. Regardless, future research should use other methods (e.g., depressive symptom and sleep difficulty scores obtained from a psychiatric diagnosis, daily sleep diaries, and actigraphy, instead of self-report), as well as a longitudinal design to reduce potential CMV biases (see Podsakoff et al. 2012). Third, as the sample was comprised of only Japanese college students, generalizations to other samples should be made with caution. Our sample in the current study was a group of young adults (*M* = 17.75, *SD* = 8.95) who had nearly the same AAQ-II mean as another age group, which consisted of employees in the United Kingdom (*M* = 18.53, *SD* = 7.52, *N* = 583, Bond et al. 2011); a *t* test of the difference between the AAQ-II means in the current study and in Bond et al.’s study (2011) was not significant (*t*(1244) = 1.65, *p* = 0.099). However, further research with different ages may contribute to understanding the association between psychological inflexibility, depressive symptoms, and insomnia. In particular, it is important to determine the generalizability to clinical populations due to the use of symptom questionnaires and the potential transient nature of the symptoms. Moreover, the current study did not assess the participant’s health status, which is a potential confounding variable and an obvious shortcoming.

Finally, although the current study measured psychological inflexibility using the AAQ-II, some studies (e.g., Wolgast 2014) have argued that psychological inflexibility as measured with this instrument overlaps with other psychological dysfunctions. However, other studies (e.g., Bryan et al. 2015; Curtiss and Klemanski 2014; Pennato et al. 2013) have provided evidence that refutes this statement. For example, AAQ-II scores were associated with the PHQ-9 scores in each of three Asian cultures (India, the Philippines, and Singapore), even when controlling for the effect of depressive symptoms as measured using the Center for Epidemiologic Studies’ Depression Scale (Kato 2016). The current study also showed that AAQ-II scores were associated with PHQ-9 scores after controlling for the effect of SDS scores, and were associated with SDS scores after controlling for the effect of PHQ-9 scores. Consequently, future research determining whether the AAQ-II has sufficient discriminant validity related to psychological dysfunction, particularly in depressive symptoms, may be useful to address this issue.

## Conclusions

Despite these limitations, the current study contributes to the greater understanding of outcomes associated with components of ACT by providing evidence that greater psychological inflexibility was linked with high levels of depressive symptoms and sleep difficulties.

## Abbreviations

AAQ: Acceptance and Action Questionnaire
ACT: acceptance and commitment therapy
CI: confidence interval
CMV: common method variance
DSM: diagnostic and statistical manual of mental disorders
NIH: National Institutes of Health
PHQ: Patient Health Questionnaire
SDS: Sleep Difficulty Scale
SEM: structural equation modeling

## References

[CR1] Alloy LB, Abramson LY, Metalsky GI, Hartlage S (1988). The hopelessness theory of depression: attributional aspects. Br J Clin Psychol.

[CR2] Baglioni C, Battagliese G, Feige B, Spiegelhalder K, Nissen C, Voderholzer U, Lombardo C, Riemann D (2011). Insomnia as a predictor of depression: a meta-analytic evaluation of longitudinal epidemiological studies. J Affect Disord.

[CR3] Baron RM, Kenny DA (1986). The moderator-mediator variable distinction in social psychological research: conceptual, strategic, and statistical considerations. J Pers Soc Psychol.

[CR4] Berk M (2009). Sleep and depression: theory and practice. Aust Fam Physician.

[CR5] Biegel GM, Brown KW, Shapiro SL, Schubert CM (2009). Mindfulness-based stress reduction for the treatment of adolescent psychiatric outpatients: a randomized clinical trial. J Consult Clin Psychol.

[CR6] Bohlmeijer ET, Fledderus M, Rokx TA, Pieterse ME (2011). Efficacy of an early intervention based on acceptance and commitment therapy for adults with depressive symptomatology: evaluation in a randomized controlled trial. Behav Res Ther.

[CR7] Bond FW, Hayes SC, Baer RA, Carpenter KM, Guenole N, Orcutt HK, Waltz T, Zettle RD (2011). Preliminary psychometric properties of the Acceptance and Action Questionnaire-II: a revised measure of psychological flexibility and experiential avoidance. Behav Ther.

[CR8] Bryan CJ, Ray-Sannerud B, Heron EA (2015). Psychological flexibility as a dimension of resilience for posttraumatic stress, depression, and risk for suicidal ideation among air force personnel. J Context Behav Sci.

[CR9] Buysse DJ, Reynolds CF, Monk TH, Berman SR, Kupfer DJ (1989). The Pittsburgh Sleep Quality Index: a new instrument for psychiatric practice and research. J Psychiatr Res.

[CR10] Catanzaro SJ, Mearns J (1990). Measuring generalized expectancies for negative mood regulation: initial scale development and implications. J Pers Assess.

[CR11] Chawla N, Ostafin B (2007). Experiential avoidance as a functional dimensional approach to psychopathology: an empirical review. J Clin Psychol.

[CR12] Cole MG, Dendukuri N (2003). Risk factors for depression among elderly community subjects: a systematic review and meta-analysis. Am J Psychiatry.

[CR13] Curtiss J, Klemanski DH (2014). Teasing apart low mindfulness: differentiating deficits in mindfulness and in psychological flexibility in predicting symptoms of generalized anxiety disorder and depression. J Affect Disord.

[CR14] Doi Y, Minowa M, Uchiyama M, Okawa M (1998). Development of the Pittsburgh Sleep Quality Index Japanese version. Jpn J Psychiatric Treat.

[CR15] Fergus TA, Valentiner DP, McGrath PB, Gier-Lonsway S, Jencius S (2013). The cognitive attentional syndrome: examining relations with mood and anxiety symptoms and distinctiveness from psychological inflexibility in a clinical sample. Psychiatry Res.

[CR16] Franzen PL, Buysse DJ (2008). Sleep disturbances and depression: risk relationships for subsequent depression and therapeutic implications. Dialogues Clin Neurosci.

[CR17] Freedman LS, Schatzkin A (1992). Sample size for studying intermediate endpoints within intervention trials of observational studies. Am J Epidemiol.

[CR18] Garland SN, Rouleau CR, Campbell T, Samuels C, Carlson LE (2015). The comparative impact of mindfulness-based cancer recovery (MBCR) and cognitive behavior therapy for insomnia (CBT-I) on sleep and mindfulness in cancer patients. Explore (NY).

[CR19] Gloster AT, Klotsche J, Chaker S, Hummel KV, Hoyer J (2011). Assessing psychological flexibility: what does it add above and beyond existing constructs?. Psychol Assess.

[CR20] Gross CR, Kreitzer MJ, Reilly-Spong M, Wall M, Winbush NY, Patterson R, Mahowald M, Cramer-Bornemann M (2011). Mindfulness-based stress reduction versus pharmacotherapy for chronic primary insomnia: a randomized controlled clinical trial. Explore (NY).

[CR21] Hayes SC, Strosahl KD, Wilson KG (1999). Acceptance and commitment therapy: an experiential approach to behavior change.

[CR23] Hayes SC, Luoma JB, Bond FW, Masuda A, Lillis J (2006). Acceptance and commitment therapy: model, processes and outcomes. Behav Res Ther.

[CR24] Heidenreich T, Tuin I, Pflug B, Michal M, Michalak J (2006). Mindfulness-based cognitive therapy for persistent insomnia: a pilot study. Psychother Psychosom.

[CR25] Hirao K (2015). Difference in mental state between Internet-addicted and non-addicted Japanese undergraduates. Int J Adolesc Med Health.

[CR26] Hu L, Bentler PM (1999). Cutoff criteria for fit indexes in covariance structure analysis: conventional criteria versus new alternatives. Struct Equ Model.

[CR27] Inagaki M, Ohtsuki T, Yonemoto N, Kawashima Y, Saitoh A, Oikawa Y, Kurosawa M, Muramatsu K, Furukawa TA, Yamada M (2013). Validity of the Patient Health Questionnaire (PHQ)-9 and PHQ-2 in general internal medicine primary care at a Japanese rural hospital: a cross-sectional study. Gen Hosp Psychiatry.

[CR28] Kämpfe CK, Gloster AT, Wittchen H, Helbig-Lang S, Lang T, Gerlach AL, Richter J, Alpers GW, Fehm L, Kircher T, Hamm AO, Ströhle A, Deckert J (2012). Experiential avoidance and anxiety sensitivity in patients with panic disorder and agoraphobia: do both constructs measure the same?. Int J Clin Health Psychol.

[CR29] Kato T (2014). Development of the Sleep Quality Questionnaire in healthy adults. J Health Psychol.

[CR30] Kato T (2014). Insomnia symptoms, depressive symptoms, and suicide ideation in Japanese white-collar employees. Int J Behav Med.

[CR31] Kato T (2016). Psychological inflexibility and depressive symptoms among Asian English speakers: a study on Indian, Philippine, and Singaporean samples. Psychiatry Res.

[CR32] Kessler RC, Bromet EJ (2013). The epidemiology of depression across cultures. Annu Rev Public Health.

[CR33] Kim K, Uchiyama M, Okawa M, Liu X, Ogihara R (2000). An epidemiological study of insomnia among Japanese general population. Sleep.

[CR34] Kroenke K, Spitzer RL, Williams JB (2001). The PHQ-9: validity of a brief depression severity measure. J Gen Int Med.

[CR35] Lundh L (2005). The role of acceptance and mindfulness in the treatment of insomnia. J Cognit Psychother.

[CR36] MacKinnon DP, Krull JL, Lockwood C (2000). Equivalence of the mediation, confounding, and suppression effect. Prev Sci.

[CR37] MacKinnon DP, Lockwood CM, Hoffman JM, West SG, Sheets V (2002). A comparison of methods to test mediation and other intervening variable effects. Psychol Methods.

[CR38] Manber R, Edinger JD, Gress JL, San Pedro-Salcedo MG, Kuo TF, Kalista T (2008). Cognitive behavioral therapy for insomnia enhances depression outcome in patients with comorbid major depressive disorder and insomnia. Sleep.

[CR39] Manea L, Gilbody S, McMillan D (2012). Optimal cut-off score for diagnosing depression with the Patient Health Questionnaire (PHQ-9): a meta-analysis. CMAJ.

[CR40] Manea L, Gilbody S, McMillan D (2015). A diagnostic meta-analysis of the Patient Health Questionnaire-9 (PHQ-9) algorithm scoring method as a screen for depression. Gen Hosp Psychiatry.

[CR41] Masuda A, Mandavia A, Tully EC (2014). The role of psychological inflexibility and mindfulness in somatization, depression, and anxiety among Asian Americans in the United States. Asian Am J Psychol.

[CR42] McCracken LM, Williams JL, Tang NKY (2011). Psychological flexibility may reduce insomnia in persons with chronic pain: a preliminary retrospective study. Pain Med.

[CR43] McCracken LM, Barker E, Chilcot J (2014). Decentering, rumination, cognitive defusion, and psychological flexibility in people with chronic pain. J Behav Med.

[CR44] Muramatsu K, Miyaoka H, Kamijima K, Muramatsu Y, Yoshida M, Otsubo T, Gejyo F (2007). The patient health questionnaire, Japanese version: validity according to the miniinternational neuropsychiatric interview-plus. Psychol Rep.

[CR45] National Institutes of Health (1998) Insomnia: assessment and management in primary care. National Institutes of Health Publications. No. 98-4088

[CR46] Nolen-Hoeksema S (1991). Responses to depression and their effects on the duration of depressive episodes. J Abnorm Psychol.

[CR47] Ong JC, Shapiro SL, Manber R (2008). Combining mindfulness meditation with cognitive-behavior therapy for insomnia: a treatment-development study. Behav Ther.

[CR48] Ong JC, Ulmer CS, Manber R (2012). Improving sleep with mindfulness and acceptance: a metacognitive model of insomnia. Behav Res Ther.

[CR49] Öst L (2008). Efficacy of the third wave of behavioral therapies: a systematic review and meta-analysis. Behav Res Ther.

[CR50] Öst L (2014). The efficacy of acceptance and commitment therapy: an updated systematic review and meta-analysis. Behav Res Ther.

[CR51] Pennato T, Berrocal C, Bernini O, Rivas T (2013). Italian version of the Acceptance and Action Questionnaire-II (AAQ-II): dimensionality, reliability, convergent and criterion validity. J Psychopathol Behav Assess.

[CR52] Podsakoff PM, MacKenzie SB, Podsakoff NP (2012). Sources of method bias in social science research and recommendations on how to control it. Annu Rev Psychol.

[CR53] Roberts RE, Duong HT (2013). Depression and insomnia among adolescents: a prospective perspective. J Affect Disord.

[CR54] Ruiz FJ (2010). A review of acceptance and commitment therapy (ACT) empirical evidence: correlational, experimental psychopathology, component and outcome studies. Int J Psychol Psychol Ther.

[CR55] Ruiz FJ (2012). Acceptance and commitment therapy versus traditional cognitive behavioral therapy: a systematic review and meta-analysis of current empirical evidence. Int J Psychol Psychol Ther.

[CR56] Schramm PJ, Zobel I, Mönch K, Schramm E, Michalak J (2016). Sleep quality changes in chronically depressed patients treated with mindfulness-based cognitive therapy or the cognitive behavioral analysis system of psychotherapy: a pilot study. Sleep Med.

[CR57] Sobel ME, Leinhardt S (1982). Asymptotic confidence intervals for indirect effects in structural models. Sociological methodology.

[CR58] Staner L (2010). Comorbidity of insomnia and depression. Sleep Med Rev.

[CR59] Taylor DJ, Lichstein KL, Weinstock J, Sanford S, Temple JR (2007). A pilot study of cognitive-behavioral therapy of insomnia in people with mild depression. Behav Ther.

[CR60] Williams JMG (2008). Mindfulness, depression and modes of mind. Cognit Ther Res.

[CR61] Winbush NY, Gross CR, Kreitzer MJ (2007). The effects of mindfulness-based stress reduction on sleep disturbance: a systematic review. Explore (NY).

[CR62] Wolgast M (2014). What does the Acceptance and Action Questionnaire (AAQ-II) really measure?. Behav Ther.

[CR63] Woodruff SC, Glass CR, Arnkoff DB, Crowley KJ, Hindman RK, Hirschhorn EW (2014). Comparing self-compassion, mindfulness, and psychological inflexibility as predictors of psychological health. Mindfulness.

[CR64] Yokoyama E, Kaneita Y, Saito Y, Uchiyama M, Matsuzaki Y, Tamaki T, Munezawa T, Ohida T (2010). Association between depression and insomnia subtypes: a longitudinal study on the elderly in Japan. Sleep.

[CR65] Yook K, Lee S, Ryu M, Kim K, Choi TK, Suh SY, Kim Y, Kim B, Kim MY, Kim M (2008). Usefulness of mindfulness-based cognitive therapy for treating insomnia in patients with anxiety disorders: a pilot study. J Nerv Ment Dis.

[CR66] Zhang C, Chung P, Si G, Liu JD (2014). Psychometric properties of the Acceptance and Action Questionnaire–II for Chinese college students and elite Chinese athletes. Meas Eval Couns Dev.

